# A diffusion MRI-based structural assessment of the striatal compartments, striosome and matrix, in Obsessive Compulsive Disorder

**DOI:** 10.21203/rs.3.rs-10646305/v1

**Published:** 2026-08-26

**Authors:** Jeff Waugh, An Tieu, Rebecca Price, Emily Stern

**Affiliations:** University of Texas Southwestern; University of Pittsburgh; Icahn School of Medicine at Mount Sinai

## Abstract

The striatum is central to habit formation and behavioral control, and striatal dysfunction has been implicated in compulsive actions and loss of inhibition. Dysregulation of inhibitory control circuitry may contribute to the repetitive, disabling behaviors observed in obsessive compulsive disorder (OCD). Striatal medium spiny projection neurons are organized within two distinct tissue compartments: the striosome forms a web-like tubular structure which spans the whole striatum and is embedded within the surrounding matrix. The compartments serve discrete functions in behavior regulation and have selective vulnerabilities in neurological diseases, motivating the investigation of the compartments in OCD. We performed a connectivity-based parcellation of the human striatum *in vivo* using diffusion MRI, identifying voxels whose relative abundance, spatial distribution, and structural connectivity profiles matched those of striosome and matrix in striatal tissue. Cortico-striate projections were organized into somatotopic zones that were distributed throughout the striatum. In OCD we found a shift from matrix-like to striosome-like volume, most abundantly in the bilateral rostral caudate and putamen, where striosome-like volume was increased by 24.3%. This shift toward striosome-like connectivity was 2.5-fold larger in caudate than in putamen. Cortico-striate projections were organized into somatotopic zones distributed throughout the striatum. Compartment bias was shifted toward striosome-like volume in 80% of these somatotopic zones. Volume did not shift uniformly throughout the compartment-like probability distribution, suggesting that increased striosome branch diameter may be a neuroanatomic underpinning of OCD. Assessing the organization and connectivity of striosome and matrix in living humans is essential for understanding the pathophysiology of OCD.

## Introduction

Obsessive compulsive disorder (OCD) affects 2–3% of adults in the United States,^1^ with symptoms commonly manifesting as obsessions (repetitive and intrusive irrational thoughts) and compulsions (repetitive behaviors or mental acts performed in response to the obsessions).^2^ The gold standard for diagnosis of OCD is through psychiatric clinical interview (e.g. Structured Clinical Interview for DSM disorders, or SCID) without the aid of biomarkers^3^ or insight into how specific structural and/or functional brain abnormalities influence the diverse behavioral deficits seen in OCD.^4^ A better understanding of the neurobiological and neuroanatomical substrates contributing to OCD may facilitate the development of clinically useful biomarkers for diagnosis and treatment, while improving our understanding of how structural brain abnormalities contribute to obsessions and compulsive behaviors.

Since the 1980s, MRI studies in OCD have identified structural and functional abnormalities in several brain regions.^5–8^ The prevailing neurobiological model of OCD implicates dysfunction of cortico-striato-thalamo-cortical (CSTC) circuitry, with the striatum playing a central role in regulating information flow from cortical regions.^9^ Consistent with its involvement in habit formation and repetitive behaviors,^10–13^ structural MRI has shown reduced striatal volume in OCD, including both caudate and putamen.^13–15^ Functional MRI has likewise demonstrated altered striatal activity during resting-state and reward processing tasks.^13,16^

The striatum is composed of two functionally distinct tissue compartments, the matrix and striosome.^17^ These compartments differ in their expression of neurochemical markers,^18^ their spatial distribution within the striatum,^19^ and their relative abundance.^19,20^ The matrix and striosome also demonstrate spatially segregated structural^21,22^ and functional networks.^23^ The compartments receive distinct cortical inputs that suggest distinct roles in behavioral regulation pertinent to OCD. Striosomes preferentially receive projections from regions suggested to be involved in reward prediction error processing and behavioral adaptation.^24–26^ In contrast, matrix receives input from regions associated with response inhibition, value-based decision-making, and motor planning.^26–28^

Functional MRI indicates that the compartments are engaged in distinct resting state^23^ and task-specific^29,30^ functional networks. Additionally, matrix and striosome show different degrees of vulnerability to degeneration and dysfunction in neuropsychiatric disorders, including Huntington disease, Parkinson disease, stimulant addiction, and schizophrenia.^31–35^ Abnormal compartment volumes were previously described in other disorders that are commonly comorbid with OCD, such as autism spectrum disorder,^36,37^ generalized anxiety disorder,^38^ and major depression.^39^ Functional differences between compartments have also been observed in movement disorders, including preferential upregulation of dopamine-related signaling in the striosome in Parkinson disease (similar findings in both non-human primates^40^ and humans^41^) and depletion of dopamine synthesis in the striosome in DOPA-responsive dystonia.^42^ Similarly, in Parkinson disease dopaminergic denervation was associated with an expansion of the matrix.^43^ Treatments specifically targeting dopamine receptor signaling in the striosome demonstrated significant benefits in reducing OCD severity.^44^ Characterizing striatal compartment abnormalities in OCD may therefore provide insight into the anatomical substrates underlying OCD-related behaviors.

To the best of our knowledge, no prior studies in humans have characterized striatal compartment abnormalities in OCD, though compartment-specific abnormalities have been proposed as a potential contributor to OCD.^45,46^ We developed a diffusion MRI method to identify striatal voxels with matrix-like and striosome-like patterns of structural connectivity *in vivo*.^26^ Using this approach, we found striatal voxels in human that mirror the connectivity biases of matrix and striosome tissue found in animal tract-tracing studies. Striatal compartments identified with our method also recapitulate key anatomical features described in human and animal tissue, including their relative volume, spatial distribution within the striatum, and connectivity biases.^19–22,26^ Our connectivity-based striatal parcellations are highly reliable (test–retest error in scans one month apart: 0.14%)^26^ and highly specific: shifting the location of striosome-like voxels by only a few millimeters abolishes compartment-specific biases in structural and functional connectivity.^22,23^ The separable functions of matrix and striosome, together with the potential for compartment-selective injury or maldevelopment to alter the ratio of matrix:striosome function, suggest that localizing abnormalities in OCD to the matrix, striosome, or both compartments could have direct implications for diagnosis and treatment. Therefore, we undertook a structural assessment of the striatal compartments in OCD and matched healthy control subjects to test the hypothesis that the ratio of striosome to matrix is abnormal in OCD.

## Methods

### MRI Acquisition

This was a secondary analysis of structural (T1) and diffusion weighted imaging data from three separate studies, all scanned at 3T. Dataset 1 comprises 85 matched pairs of OCD and HC (healthy control) subjects obtained in two NIH funded studies (R01MH111794 and R33MH107589; non-diffusion data from this sample are reported elsewhere^47–51^). All individuals in this cohort had either a DSM-5 diagnosis of OCD (for patient subjects) or an absence of any DSM-5 Axis I disorder (for HC subjects) confirmed by a trained clinical rater as part of the study prior to their enrollment. Dataset 2 consisted of 156 subjects with obsessive-compulsive and related disorders recruited into a larger NIH-funded clinical trial (NCT04580043 funded by R01MH124707), as described in a previous interim report.^52^ Of these, 138 individuals had a formal prior diagnosis of OCD, while 18 met study inclusion criteria based on the presence of other compulsive behavior disorders. The exclusion of these 18 subjects did not meaningfully or significantly alter any OCD–HC comparisons. Dataset 2 utilized the diffusion MRI protocol from the Human Connectome Project (HCP).^53^ Therefore, we matched Dataset 2 patient subjects to HC subjects from the HCP. OCD and HC subjects were matched for diffusion MRI protocol, sex and age (± 3 years) in all studies. Comparisons of MRI measures between OCD and HC subjects were subsequently performed in a group-wise manner. Dataset 1 diffusion MRI was collected at a resolution of 2 mm isotropic, while Dataset 2 and HCP diffusion MRI scans were collected at 1.5 mm isotropic. Structural T1 MRI for both Dataset 1 and 2 were collected at 0.80 mm isotropic.

### MRI Processing

Diffusion MRI scans were processed using the FSL FMRIB Diffusion Toolbox with default parameters.^54^ Brain extraction was performed with *bet2*, and eddy current distortions and motion artifacts were corrected using *eddy_cuda10.2*.^55^ Diffusion tensors were fitted at each voxel using *dtifit*. To model crossing fibers and estimate voxel-wise diffusion probability distributions, we used *bedpostx_gpu*.^56^ We defined regions of interest for striatal parcellation in the MNI152_T1_1mm standard space using the Talairach atlas^57^ and registered standard space masks into each subject’s native diffusion space using *flirt* and *fnirt*.

We used Freesurfer’s anatomical segmentation tool for structural MRI, *recon-all*, to perform volumetric segmentation, extracting the brainmask volume, which includes the summed volume of gray matter, white matter, and cerebrospinal fluid.^58^ We used brainmask to normalize for differences in intracranial volume when characterizing nucleus size differences.^59^

### Identifying Matrix-like and Striosome-like Voxels

Data analyses proceeded in two major steps. First, we identified voxels in the dorsal striatum (caudate and putamen) that exhibited structural connectivity with extra-striate regions shown to preferentially connect to matrix or striosome compartments in prior studies.^26^ Second, once we identified these “matrix-like” or “striosome-like” voxels (using the term “like” to distinguish our inferential parcellations from direct identification of matrix and striosome through histochemical staining), we calculated the relative volume of these between OCD and HC groups.

Our striatal mask included the caudate and putamen but excluded the posterior half of the caudate tail, as this region contains very little striosome^60^ and its narrow structure in the coronal plane reduces registration accuracy.^26^ We excluded the nucleus accumbens due to the absence of prior tract-tracing studies to guide the selection of matrix- and striosome-favoring bait regions. We performed connectivity-based parcellation^61^ of the striatum using masks of extra-striate target regions that selectively project to either the matrix or striosome compartments.^21,26^ The matrix target mask included the inferior frontal gyrus pars opercularis, primary motor cortex, supplementary motor area, primary somatosensory cortex (Brodmann Areas 1, 2, and 3), and the superior parietal cortex. The striosome target mask included the posterior orbitofrontal cortex, anterior insula, basolateral amygdala, basal operculum, and posterior temporal fusiform cortex. Extra-striate regions with biases toward one compartment can include voxels with connectivity to the other compartment.^62^ Additionally, a region may favor one compartment within a specific striatal somatotopic zone but lose that bias in other striatal areas.^23,26,63^ Therefore, we included all bait region targets into composite matrix-favoring and striosome-favoring masks to minimize the intrastriate somatotopic variance in compartment connectivity.

We performed tractography using FSL *probtrackx2_gpu*^56^ in classification targets mode with the following parameters: curvature threshold = 0.2; steplength = 0.5 mm; 2,000 steps per sample; 5,000 streamlines per seed voxel, setting striatal voxels as the seed and the matrix- or striosome-favoring bait regions as targets. All rounds of tractography were performed in native diffusion space and separately in each hemisphere. We utilized the FSL tool *proj_thresh* to calculate the voxelwise probability of connecting to matrix-favoring or striosome-favoring target extra-striate masks. This approach generates two superimposable probability maps, one for the matrix-like and one for the striosome-like distribution, each with P = 0–1. At each striatal voxel, the sum of these two probabilities was always 1. We classified each striatal voxel as matrix-like, striosome-like, or indeterminant based on how its structural connectivity compared with the connectivity biases described for matrix and striosome in tissue, as defined by the ratio of streamlines that connected to matrix- or striosome-favoring bait masks. Within each distribution, we defined voxels with a connection probability of P ≥ 0.55 as reaching bias threshold, and thus meeting criteria to be defined as matrix-like or striosome-like. We chose this threshold to capture the full range of compartment-like bias (from 0.55-1.0) and for consistency with prior disease compartment investigations.^37–39^ Voxels between the lower limits of the compartment-like distributions (P = 0.45–0.55) were defined as indeterminate. We extracted matrix-like and striosome-like volume and normalized each by the total compartment-like volume (matrix + striosome). We quantified the volume of matrix-like and striosome-like voxels separately in caudate and putamen. Across the full range of bias thresholds (0.55 < P < 1.0), the matrix-like and striosome-like voxels consistently replicated characteristics of matrix and striosome defined through histology, such as the relative abundance, spatial distribution, and extra-striate connectivity.^21–23,37–39^

Since our parcellation method relies on differential connectivity, differences in compartment-like volume could result from several types of tissue-level abnormality (e.g., decreased matrix tissue and increased striosome tissue could both result in an increase in striosome-like volume). However, each type of striatal tissue abnormality can influence compartment-like volume differently at distinct portions of the probability distribution.^37^ To better understand the tissue-level abnormalities which may underlie compartment volume differences between OCD and HC groups, we extracted volume in a histogram that spanned the probability distribution (P = 0–1, span: 0.02 per bin). Due to marked differences in the total matrix- and striosome-like volume, we transformed (natural log) the volume data to aid in visualization and comparison.

### The Influence of Each Compartment-favoring Bait Region

To identify the influence of each extra-striate bait region on compartment-like volume, we performed 10 subsequent rounds of probabilistic tractography, each using only nine bait regions, rotating the left-out bait region for each round. These 10 “N-1” parcellations allowed us to quantify connectivity with the omitted region and to map the somatotopic zones of the striatum that were most influenced by each bait region. We subtracted each “N-1” parcellation from the original (all 10 bait regions) parcellation; the differences between these parcellations quantified the influence of the left-out bait region at a voxelwise level. We thresholded these difference maps (using *fslmaths*) to ensure that each somatotopic zone was distinct (no overlap between multiple somatotopic zones), and that each somatotopic zone was similarly sized. We then quantified compartment-like volumes (P ≥ 0.55) within each of these 10 somatotopic zones.

### Statistical Tests

We performed two-tailed, two-sample t-tests with unequal variance to assess significance in measures in a group-wise manner across OCD and HC subjects. We corrected for multiple comparisons using the family-wise error (FWE) method of Benjamini and Hochberg.^64^ We defined the following families of tests for these FWE corrections: Compartment-like volumes between OCD and HC groups (4 tests), Histogram analysis of compartment bias (50 tests), and Somatotopic zone compartment-like volumes (10 tests).

In addition to these family-specific t-tests, we used the FSL tool *randomise* to perform voxelwise nonparametric permutation inference testing of the whole striatum to identify voxels with significant differences in compartment-like bias between OCD and HC subjects. While we generated tractography in each hemisphere independently, we combined the hemispheres for each subject prior to *randomise* testing to reduce the number of comparisons. We performed *randomise* with 5,000 permutations, 2 mm variance smoothing, threshold-free cluster enhancement, and masked by the same striatal mask utilized as the seed for tractography.

## Results

### Experimental Cohort

This study included 526 subjects (263 matched OCD-HC pairs), including 346 females and 180 males, with an average age of 30 years (range: 17–62 years).

### Whole Brain, Caudate, and Putamen Measures

Brain volume in OCD subjects was 8.9% smaller than in healthy controls (OCD: 1,573,285 mm^3^ vs HC: 1,444,999 mm^3^, p = 2.8x10^− 16^). We then assessed caudate and putamen volume from each subject’s diffusion space masks, normalized by the brainmask volume. Normalized volume did not differ between OCD and HC for either nucleus: caudate (OCD: 0.00206 vs HC: 0.00211, p = 0.11); putamen (OCD: 0.00245 vs HC: 0.00249, p = 0.06). Mean diffusivity for the dorsal striatum did not differ between groups (OCD: 5.9x10^− 4^ vs. HC: 5.9x10^− 4^, p = 0.81).

### OCD Subjects Exhibit Lower Matrix-like and Higher Striosome-like Volumes Than HCs

**Figure 1:**
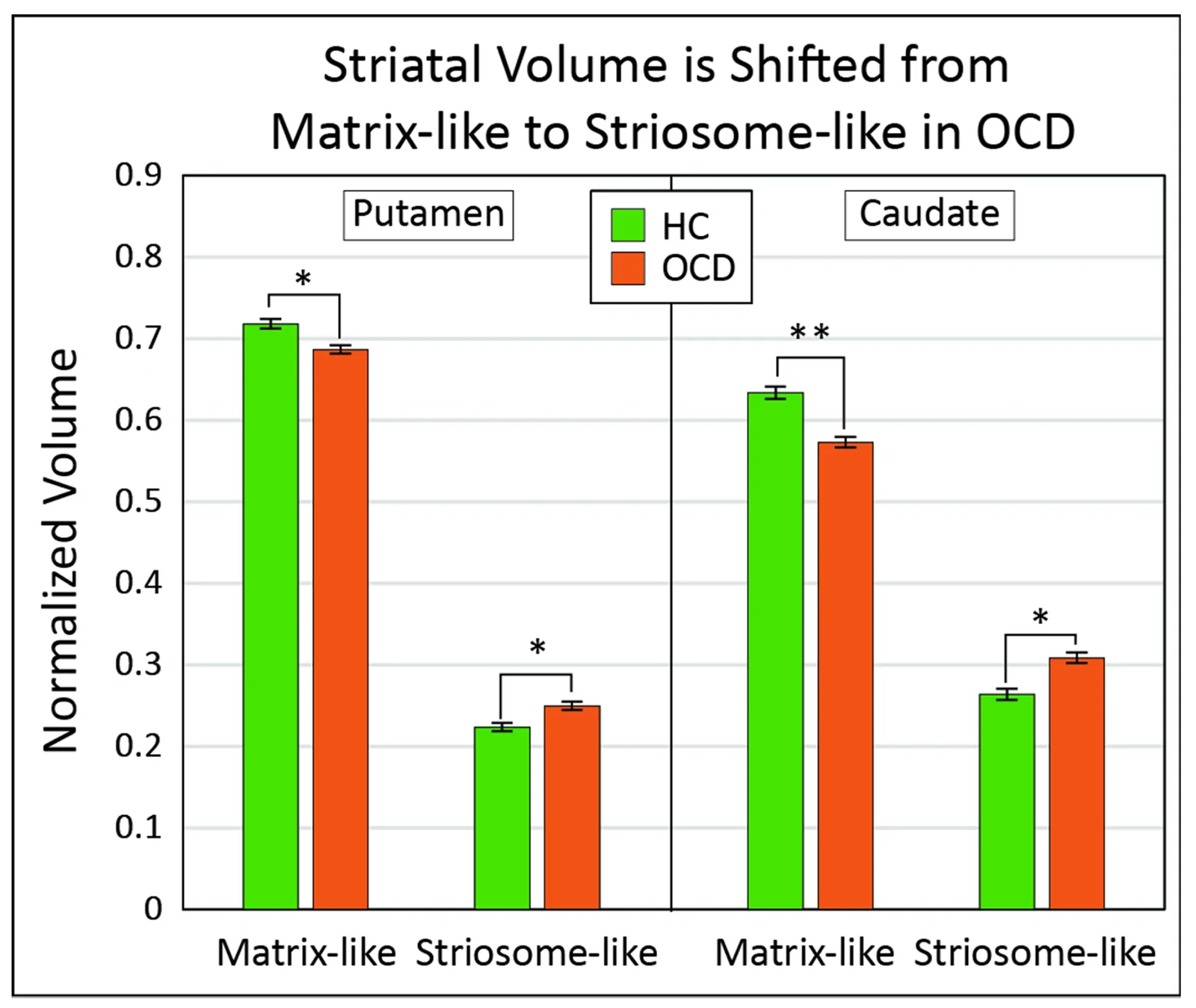
Obsessive Compulsive Disorder (OCD) subjects exhibited reduced matrix-like volume and increased striosome-like volume in both the caudate and putamen relative to matched healthy controls (HC). In the putamen, OCD subjects showed 9.6% lower matrix-like volume and 17.0% higher striosome-like volume compared to controls. In the caudate, matrix-like volume was 4.4% smaller in OCD relative to controls, whereas striosome-like volume was 11.7% larger. Volumes were normalized within each striatal nucleus and expressed as proportions of total compartment volume (matrix + striosome). Error bars represent the standard error of the mean. *, p<4.0x10^−4^. **, p=1.4x10^−9^. Significance testing corrected for multiple comparisons.

We expressed compartment-like volume as the relative abundance (striosome-like or matrix-like volume divided by the total compartment-like volume [striosome-like plus matrix-like]). Compared to HC, OCD subjects had increased striosome-like volume and decreased matrix-like volume in both putamen and caudate (Fig. 1). In the putamen, OCD subjects had 11.7% larger normalized striosome-like volume (p = 4.0x10^− 4^) and 4.4% smaller normalized matrix-like volume (p = 3.9x10^− 5^) relative to HCs. Raw volume measures in the putamen also indicated a shift from matrix- to striosome-like in the OCD putamen: striosome, OCD: 940 mm^3^ vs. HC: 859 mm^3^; matrix, OCD: 2630 mm^3^ vs. HC: 2799 mm^3^. Raw indeterminate volume (voxels with weak or no compartment-like bias, P = 0.45–0.55) in the putamen were 7.1% lower in OCD (OCD: 154 mm^3^ vs. HC 166 mm^3^, p = 3.0x10^− 3^).

In the caudate, OCD subjects had 17.1% greater striosome-like volume (p = 2.8x10^− 6^) and 9.6% less matrix-like volume (p = 1.4x10^− 9^) than HCs. Raw compartment volume measures in the caudate also mirrored the shift from matrix- to striosome-like in OCD: striosome, OCD: 988 mm^3^ vs. HC: 831 mm^3^; matrix, OCD: 2045 mm^3^ vs. HC: 1884 mm^3^. Raw indeterminate volume in the caudate was 8.9% lower in OCD than HC (OCD: 204 mm^3^ vs. HC 224 mm^3^, p = 1.0x10^− 4^).

Whole-striatum measures paralleled the differences noted for putamen and caudate. OCD subjects had 14.4% larger normalized striosome-like volume (p = 9.5x10^− 7^) and 6.7% smaller normalized matrix-like volume (p = 9.9x10^− 10^) than HCs. Raw volume measures in the whole-striatum paralleled the relative volume differences: striosome, OCD: 1928 mm^3^ vs. HC: 1690 mm^3^; matrix, OCD: 4514 mm^3^ vs. HC: 4844 mm^3^.

### The Shifted Compartment-like Bias in OCD Localizes to the Rostral Striatum

**Figure 2:**
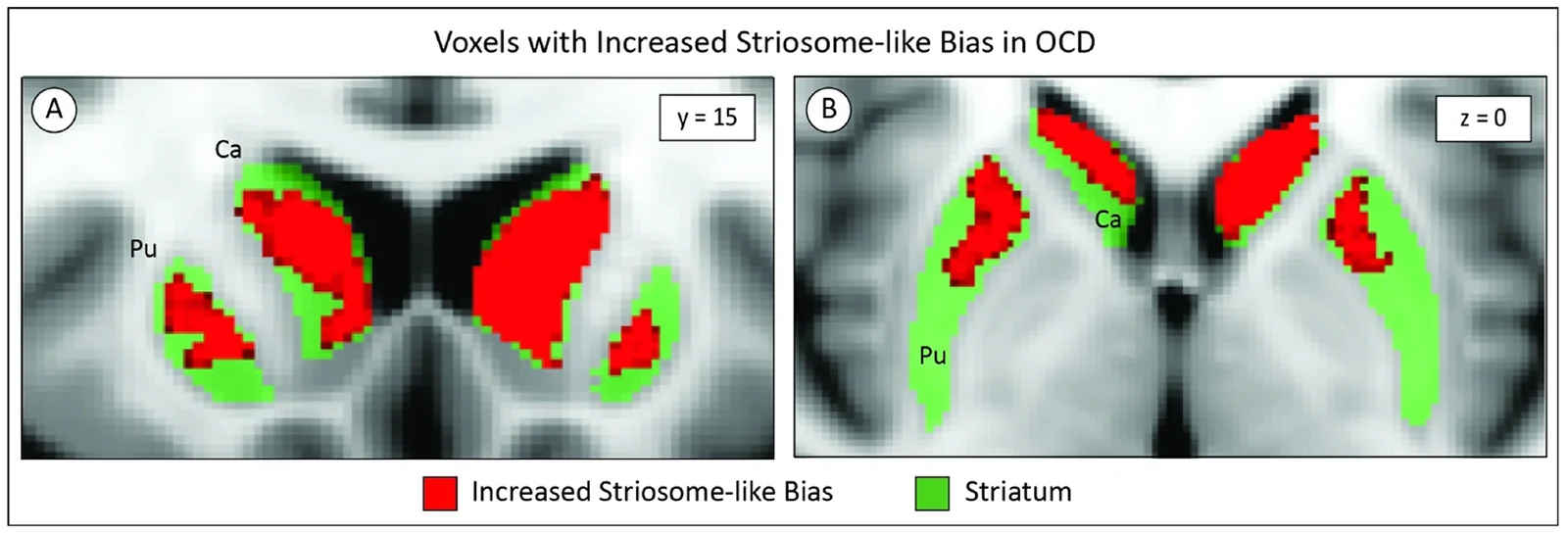
In OCD subjects, compartment bias was shifted from matrix-like to striosome-like, most abundantly in discrete clusters within the rostral striatum, as shown in the coronal (A) and axial (B) views. Voxelwise assessment of compartment-like bias (using FSL’s *randomise*) identified voxels in the bilateral rostral caudate (Ca) and the bilateral rostral putamen (Pu) where bias was significantly shifted towards greater striosome-like connectivity (red) in OCD subjects. Significance threshold, p< 0.05, FWE-corrected for multiple comparisons within *randomise*. The images adhere to radiographic convention and coordinates follow MNI convention.

We used FSL’s voxelwise nonparametric comparison tool, *randomise*,^65^ to localize these significant shifts in compartment bias to specific areas of the striatum (Fig. 2). Discrete regions of the bilateral rostral striatum, in both caudate and putamen, exhibited a shift from matrix-like bias to striosome-like bias. The volume of caudate with a shift in bias was 3958 mm^3^ (42% of total caudate volume). In the putamen, 1769 mm^3^ (17% of total putaminal volume) of volume was shifted in bias. There were no striatal voxels that shifted towards increased matrix-like bias in OCD. We extracted voxels that met minimum threshold for compartment bias (P > 0.55) within these significant regions. Relative to HCs, OCD subjects exhibited 33.3% lower matrix-like volume (p = 3.4x10^− 17^) and 24.3% greater striosome-like volume (p = 4.8x10^− 9^) within these significant regions. Mean diffusivity within the *randomise*-identified regions was similar between groups (OCD: 6.2x10^− 4^ vs. HC: 6.2x10^− 4^, p = 0.84).

### Somatotopic Organization of Projections from the Extra-Striate Bait Regions

**Figure 3:**
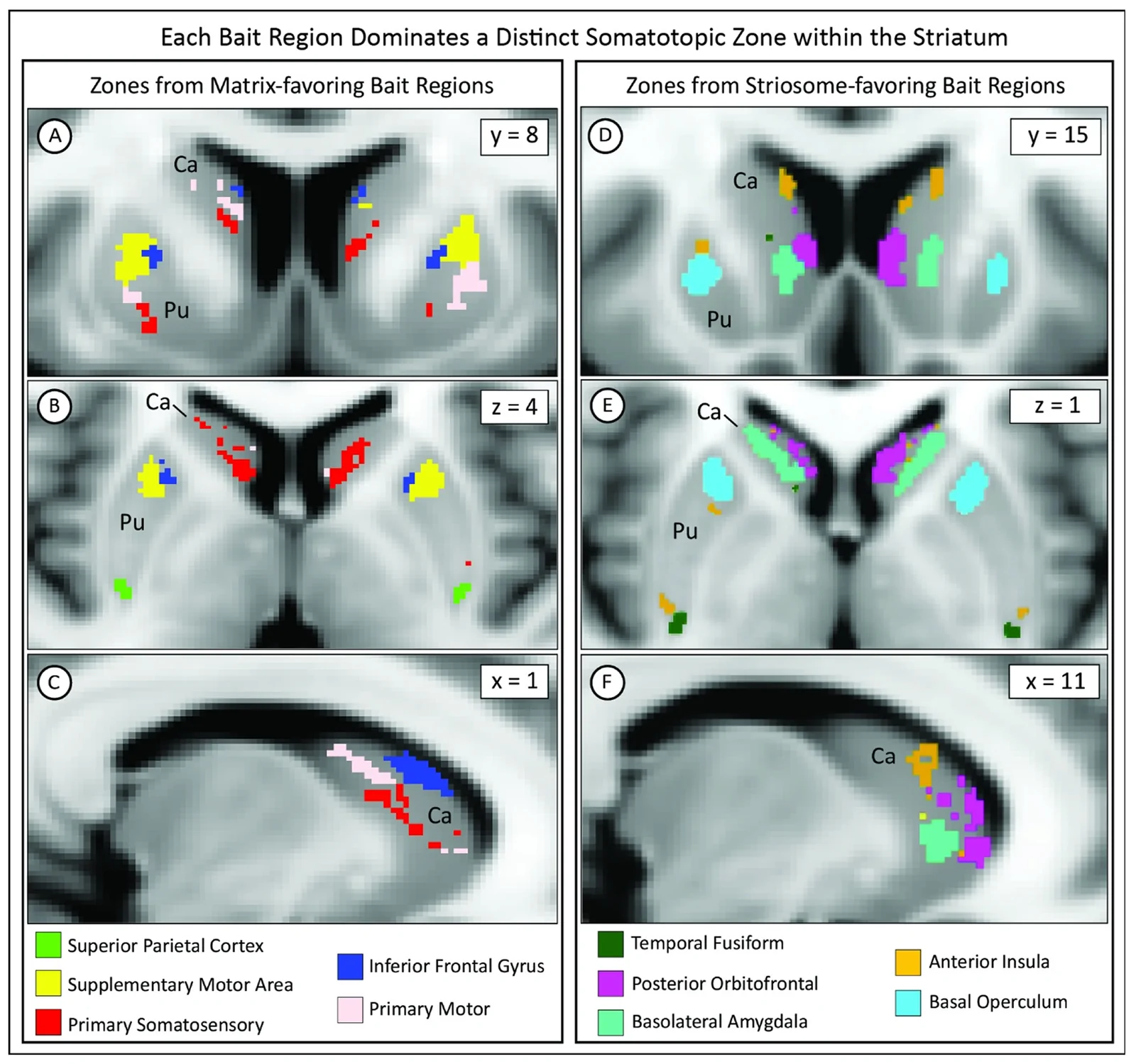
While each bait region can influence compartment bias throughout the striatum, each region dominated compartment-like bias in a distinct somatotopic zone, as seen in the coronal (A, D), axial (B, E), and sagittal (C, F) planes. Zones influenced by matrix-favoring bait regions are shown in (A–C), while zones influenced by striosome-favoring bait regions are shown in (D–F). Zones might share a border but never overlapped. Although we parcellated the left and right hemispheres independently, the location of each somatotopic zone was highly similar between hemispheres. All zones had equal volume. Pu, putamen; Ca, caudate. The images adhere to radiographic convention. Coordinates follow MNI convention.

**Figure 4:**
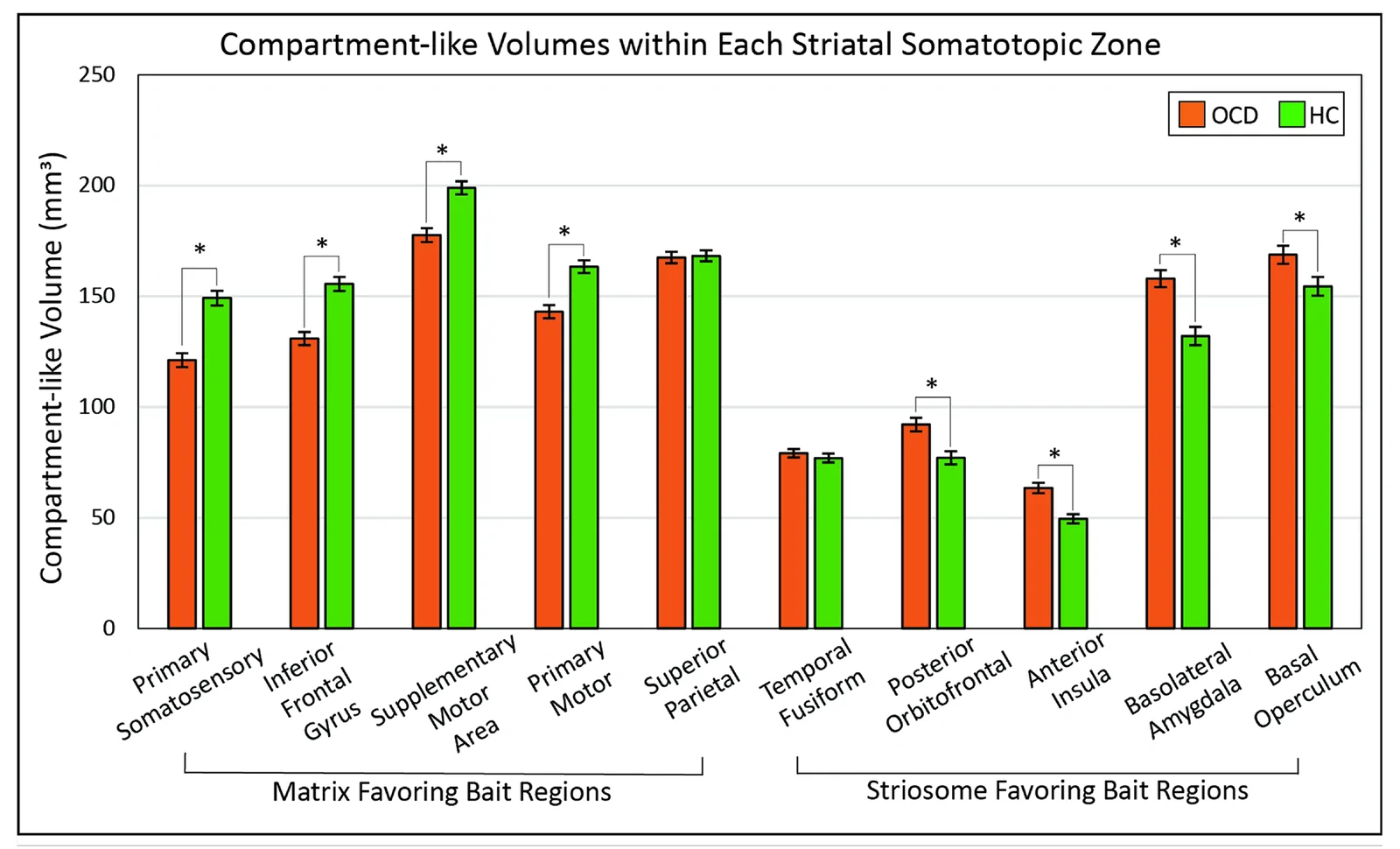
The shift from matrix-like to striosome-like volume in OCD is recapitulated in the compartment volumes extracted from the somatotopic zones (identified in figure 3) for 80% of bait regions. We assessed compartment-like volume within each somatotopic zone, comparing obsessive compulsive disorder (OCD) and healthy control (HC) subjects. Error bars represent the standard error of the mean. *, p<0.05. **, p<10^−4^. FWE-corrected for multiple comparisons.

Cortico-striate projections are somatotopically organized.^66,67^ We isolated the contribution of each compartment-favoring extra-striate bait region to compartment-like bias calculated in striatal regions and localized the somatotopic zones where each bait region was the largest contributor to this bias (Fig. 3). We measured matrix-like or striosome-like volume (P ≥ 0.55) within each somatotopic zone: matrix-like volume in the zones of matrix-favoring bait regions, and striosome-like volume in the zones of striosome-favoring bait regions (Fig. 4). We observed reduced matrix-like volume in OCD in four of five matrix-favoring zones (inferior frontal gyrus pars opercularis, primary motor cortex, supplementary motor area, primary somatosensory cortex) and increased striosome-like volume in OCD in four of five striosome-favoring zones (anterior insula, basolateral amygdala, basal operculum, posterior temporal fusiform cortex). The two somatotopic zones that did not differ between OCD and HC in volume were those in the caudal-most putamen: the superior parietal and temporal fusiform cortices. This aligns with our finding that increased striosome-like volume in OCD was concentrated in the rostral striatum (Fig. 2). The shifts in compartment-like volume within these somatotopic zones matched the overall pattern of volume shifts described at the whole-striatum level (Fig. 1).

### Histogram Analysis of Compartment Bias

**Figure 5:**
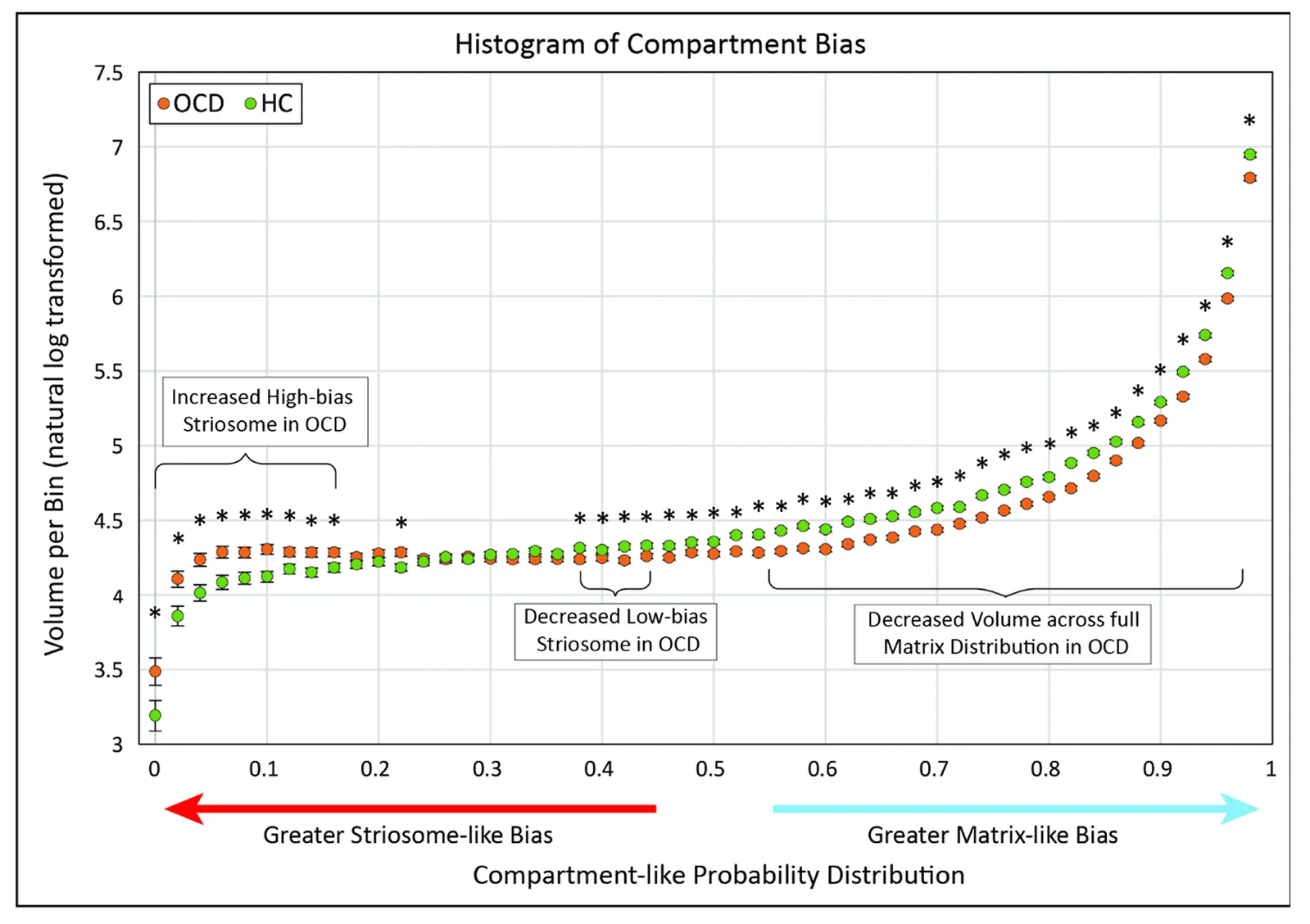
By segregating volume measures into specific bins along the compartment-like bias distribution, one can infer the nature of the tissue-level changes that underpin group-level differences in compartment-like volume. We extracted compartment-like volume in discrete bins that spanned the full probability distribution (50 bins from P=0 to P=1, bin width = 0.02), localizing volume differences between obsessive-compulsive disorder (OCD) and healthy controls (HC) to specific ranges. The leftmost bins (P=0-0.45) included striosome-like voxels while the rightmost bins (P=0.55-1.0) included matrix-like voxels. Voxels with P=0.45-0.55 were considered to be indeterminate, reflecting a weak or no bias toward either compartment. We transformed (natural log) volume measures to aid in visualization. Error bars represent the standard error of the mean. *, p<0.05. †, p<10^−6^. Significance thresholds corrected for multiple comparisons.

We extracted compartment-like volume in a histogram to identify the probability ranges in which volume was shifted in OCD (Fig. 5). OCD subjects had increased volume in the highest- and high-bias striosome-like bins (P = 0-0.16), decreased volume in low-bias striosome-like bins (P = 0.38–0.45), and reduced volume across the indeterminate and the full matrix-like distribution (P = 0.45-1.0). The highest-bias striosome-like bin was increased by 33.3% in OCD (OCD: 32 mm^3^ vs. HC: 24 mm^3^, p = 0.032). The highest-bias matrix-like bin was decreased by 14.5% in OCD (OCD: 889 mm^3^ vs. HC: 1039 mm^3^, p = 7.6x10^− 10^).

## Discussion

Prior meta-analyses of volumetric changes in the OCD striatum yielded conflicting results.^68,69^ However, the relative abundance of striosome and matrix tissue are not assessed by measures of gross striatal volume. Because striosome and matrix participate in largely segregated cortico-striatal circuits involved in action selection and behavioral inhibition, a shift in the balance between the compartments may contribute to the behavioral manifestations of OCD. For instance, the posterior medial prefrontal cortex (mPFC), which projects primarily to the striosome,^26,67^ is involved in reward prediction error processing and behavioral adaptation.^24,25^ mPFC consistently has abnormal activity patterns in both the region itself and in its network. partners in OCD.^70^ In contrast, the matrix compartment receives robust projections from lateral prefrontal regions associated with behavioral control, including the inferior frontal cortex and lateral orbitofrontal cortex,^26^ which are linked to response inhibition and value-based decision-making.^71,72^ Additionally, Brodmann Area 6, which is among the most-biased matrix-favoring cortical regions,^26,73^ contains the premotor and supplementary motor areas. Brain activity in these BA6 subregions correlates with periods of behavioral inhibition and reduced impulsivity^27^ and direct stimulation of these regions has been shown to improve response inhibition.^28^ Consistent with the functional segregation between compartments, chronic stimulant (amphetamine) exposure prolongs and enhances activity in the striosome while suppressing activity in matrix, a shift associated with the transition from goal-directed to impulse driven-habitual behavior.^74^ Therefore, the increased striosome-like to matrix-like volume ratio observed in OCD may reflect a compartment-level abnormality of the cortico-striatal circuits important in OCD.

Brimblecombe and Cragg proposed that the compartmental organization of the striatum underlies a behavioral learning schema in which interactions between the compartments promote or suppress action,^17^ and in which cholinergic interneurons located at the striosome-matrix interface^60^ facilitate action via information transfer from striosome into matrix.^75^ Conversely, when an action is deemed inappropriate and is to be suppressed, information exchange between the compartments is reduced and the action is inhibited. This model of action promotion and inhibition is supported by the observation that habitual movements (stereotypies) can be induced by upregulating the formation of cholinergic interneurons. This overexpression of cholinergic interneurons may cause an increase in information exchange between the compartments leading to the generation of habitual movement.^76^ Additionally, the selective ablation of striosome, and thus the information flow from striosome to matrix, prevents the development of cocaine-induced stereotypies.^77^ Shifting striatal volume from matrix to striosome may correlate with increased information exchange between compartments, resulting in greater facilitation of actions and persistence of striatal engrams.^78^

Functional MRI in humans has found both increased resting state activity^79,80^ and decreased activation during executive function tasks^81^ in the OCD striatum. A range of cortical effectors (including precuneus, middle cingulate, supplementary motor area, and superior frontal gyrus) have increased influence on functional activation in the rostral striatum,^82^ in areas that closely approximate the areas of expanded striosome-like volume we identified (Fig. 2). However, the functional activity of the striatal compartments has not previously been described in OCD. The compartments are differentially involved in distinct phases of action preparation and execution. The striosome exhibits greater functional activation during the cue and preparation phases of motor and cognitive tasks, while matrix activation dominates during the execution phase of tasks.^29,30^ In mice, abrupt drops in striosomal activity are associated with termination of movement,^83^ suggesting that dysregulated striosome activity may impair transitions between activity states. Such impairments could contribute to the persistent thoughts and repetitive behaviors that characterize OCD.

The control of mood and behaviors are conventionally thought to be modulated by the opposing influences of the striatal direct and indirect pathways.^84–86^ In mice, hyperactivity of indirect-pathway neurons is associated with compulsive grooming, and inhibition of these neurons reduces compulsive behavior.^87^ However, recent evidence suggests that the function of direct and indirect pathway neurons depend on their compartmental identity: both direct and indirect pathway neurons exist within both striatal compartments, but striosomal direct-indirect functions are the inverse of those arising from matrix.^88^ The canonical direct-indirect model appears to representation only matrix-derived projections. Future studies will be needed to determine how the striosome-matrix organization of the striatum interplays with the direct-indirect pathway framework in OCD.

Our findings have several important limitations. Connectivity-based striatal parcellation relies on probabilistic tractography, an inferential technique that cannot distinguish afferent from efferent projections, is susceptible to false positive connectivity, and is substantially impacted by differing acquisition parameters, all which may influence our measures of compartment-like bias.^89^ In addition, our striatal parcellation method uses extra-striate bait regions identified by injected tract tracers in animals, connectivity patterns that may be different in humans. Another limitation is that the resolution of our diffusion voxels (1.5 or 2 mm isotropic) was larger than the maximum diameter of striosome branches, as determined in histology (approximately 1.25 mm).^19,20^ This limitation assures that even highly biased striosome-like voxels will contain some matrix tissue. Higher-resolution diffusion MRI studies could distinguish matrix-like and striosome-like voxels with more granularity, potentially identifying voxels that included only matrix or only striosome tissue.

**Figure 6:**
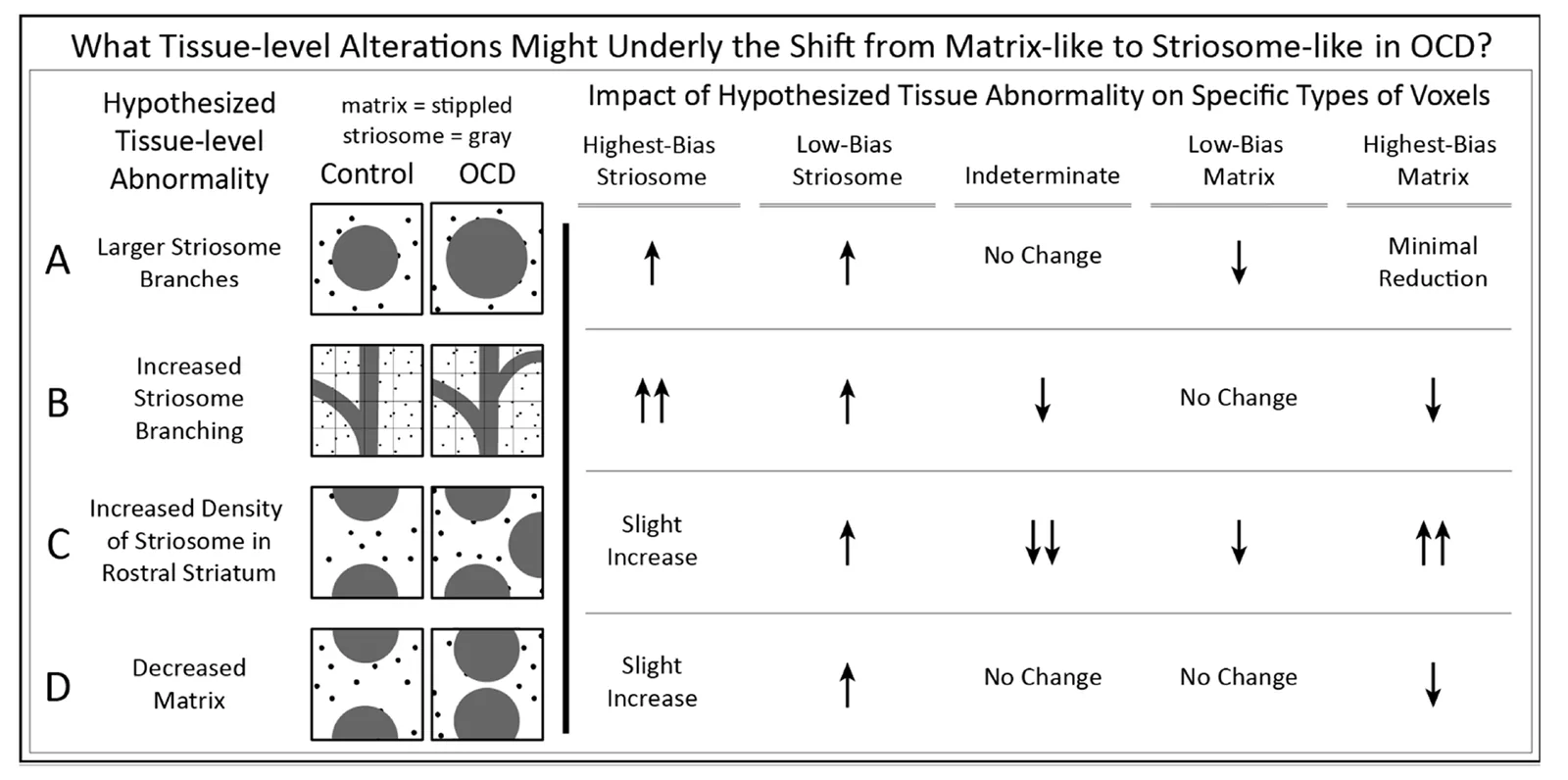
We posit that the observed shift from matrix-like to striosome-like volume in Obsessive-Compulsive Disorder (OCD) arises from specific microstructural abnormalities in striatal tissue. We describe four potential striatal compartment abnormalities that could explain this shift (A-D). Each tissue-level change will have predictable impacts on volume at each of five points on the compartment-like probability distribution: highest-bias striosome-like voxels; low-bias striosome-like voxels; indeterminate voxels (no clear bias toward either compartment); low-bias matrix-like voxels; highest-bias matrix-like voxels. Up and down arrows indicate an increase or decrease, respectively, in volume in OCD subjects relative to matched controls. Double arrows indicate a relatively larger increase or decrease. We performed a histogram analysis (Figure 5) to identify which of these hypothesized tissue abnormalities best fit the distribution of compartment-like volume in OCD.

Finally, our analyses lack a key explanatory factor: the tissue-level change(s) that produced the identified shifts in compartment-like volume. Striatal mean diffusivity did not differ between OCD and HC, suggesting that our findings were not the result of striatal injury or dysmorphisms. However, abnormal compartment development could underlie these shifts in volume (Fig. 6). The striosome is a three-dimensional, web-like structure that spreads within the matrix. Striosome branches are smaller than the resolution of our diffusion MRI voxels, so each striatal voxel includes a mixture of striosome and matrix tissue, or matrix alone. Compartment-like bias of each diffusion voxel reflects the relative amount of striosome and matrix tissue it samples. Voxels centered on striosomal branches (“bullseyes”) exhibit the highest striosome-like bias, whereas voxels that cut through one or more striosomal branches exhibit a range of biases, from low-bias striosome, to indeterminate, to low-bias matrix. Highest-bias matrix-like voxels include only matrix tissue. Therefore, localizing the shifts in OCD-related volume to particular parts of the probability distribution (Fig. 5) allows us to infer the type(s) of tissue change that led to shifts in compartment-like volume.

We evaluated four hypothesized structural abnormalities and outlined the corresponding redistribution in compartment-like bias expected for each abnormality. In each scenario, we assumed that the opposite compartment was not altered relative to control subjects. The first proposed tissue abnormality is an increase in the diameter of the striosomal branches (Fig. 6A). Voxels centered on the largest striosome branches would not change – they were already in the highest-bias striosome bin. However, increasing the diameter of moderately sized striosome branches could shift a voxel from high- to the highest-bias striosome-like bin, increasing the volume of the highest-bias striosome-like bin. Larger striosome branches would lead to increases in volume in bins throughout the striosome-like probability distribution. The volume of indeterminate voxels (those without compartment-like bias, P = 0.45–0.55) would not change, as increases in branch diameter would equally shift voxels into and out of this category (fewer low-bias matrix-like, more low-bias striosome-like voxels). Bins that sampled low-to-moderate matrix-like bias would shift to lower volume throughout the distribution, as every type of voxel-to-striosome contact – sampling of small branches, bisections, and glancing contacts – would make up a larger share of the volume in matrix-like voxels. Volume in the highest-bias matrix-like bin would decrease slightly. Such voxels, which include only matrix tissue, would be impacted only if expanded striosome branches from neighboring voxels encroached upon them, reducing their matrix-like bias.

The second proposed tissue abnormality is an increase in the number of striosome branches (Fig. 6B). Volume in the highest-bias striosome-like voxels would substantially increase as more branches would be available for bullseyes. Voxels would encounter more branches throughout the probability distribution, increasing all types of partial contact with a striosome branch. This would shift volume from the highest- and high-bias matrix-like bins downward into low-bias matrix, indeterminate, and low- and moderate-bias striosome-like bins (from right to left in Fig. 5). Since all types of branch contact would be increased in proportion to the number of branches, the volume shift toward the center of the distribution would enrich these bins to a similar, modest degree.

The third proposed tissue abnormality is a change in the likelihood that a voxel will be counted as striosome-like, without changing the volume of the striosome: an increase in spatial segregation, packing the striosome into the rostral striatum (Fig. 6C). This hypothetical change could arise from abnormalities in migration in early embryogenesis, with more striosome progenitors arriving in or spreading within the rostral striatum. Greater compartment segregation would reduce striosome-matrix contact and mixed-compartment sampling, shifting volume from the center of the distribution (low-bias striosome, indeterminate, and low-bias matrix) to high-bias striosome-like and matrix-like bins. The largest shifts in volume would occur at the highest-bias matrix bin (as more voxels would sample only matrix) and in indeterminate bins (as segregation would reduce the number of voxels that encountered both compartments in similar proportions).

The fourth proposed tissue abnormality is a decrease in matrix tissue, increasing the measure of striosome-like volume by reducing the separation between striosome branches (Fig. 6D). Since the volume and architecture of the striosome are unchanged, voxels that sampled single striosome branches would not be shifted – each would sample the same ratio of striosome:matrix tissue seen in control striata. Voxels that made partial cuts through a branch, sampling only a small volume of striosome, would remain in high-bias matrix-like bins. In contrast, voxels that made partial cuts through several branches would include less interposed matrix, decreasing the separation between branches and thus increasing striosome density and striosome-like volume without increasing total striosome volume. With the assumption that this decrease in matrix tissue is distributed uniformly throughout the striatum, thus increasing the proportion of striosome tissue uniformly across all types of partial striosome contact, volume would shift out of high- and moderate-bias matrix-like bins into low-bias matrix and indeterminate bins. However, voxels that were previously low-bias matrix or indeterminate would also shift toward higher striosome:matrix ratios, shifting volume into low-bias striosome-like bins. Therefore, volume in indeterminate and low-bias matrix-like bins would not change. With the decrease in interposed matrix tissue a small number of high-bias striosome-like voxels could add a striosome contact and shift into the highest-bias striosome-like bins. Volume in the highest-bias matrix-like bin would decrease in proportion to the decrease in matrix tissue throughout the striatum.

This pattern of volume change in Fig. 5 does not perfectly match any of the tissue changes we hypothesized, but is closest to pattern A, an increase in the diameter of striosome branches. It is possible that multiple tissue-level abnormalities could sum to produce the volume shifts we identified in OCD, or that a tissue-level abnormality is inhomogeneous across the striatum. Quantitative assessment of the striatal compartments using histology will be imperative to characterize potential striatal microstructural abnormalities in OCD.

OCD has a complex etiology, involving genetic, environmental, and neurodevelopmental factors. This complexity highlights the need to identify the anatomical abnormalities that contribute to its pathophysiology. The striatum plays a central role in motivation, executive function, and reward processing, domains that are often disrupted in OCD. However, due to the difficulty of obtaining histological data from large numbers of subjects, the striatal compartments have remained largely uncharacterized in this disorder. Our findings suggest that the balance between the striatal compartments is shifted toward striosome in OCD. Given that the striosome directly inhibits nigral dopaminergic neurons,^18,90^ leading to abrupt inhibitory pauses in dopamine release that appear to trigger behavioral transitions,^83^ these findings may have implications for dopaminergic dysfunction in OCD. Notably, medications that reduce post-synaptic dopamine signaling are commonly used in treatment-resistant OCD, suggesting that altered striosome-matrix organization may contribute to the therapeutic utility of dopamine modulation in OCD. Characterizing compartment-specific abnormalities may provide new insights into the neuroanatomic mechanisms underlying OCD-related behaviors and suggest approaches to improve its treatment.

## Data Availability

The code, bait, seed, and exclusion masks necessary to complete striatal parcellation can be accessed here: github.com/jeff-waugh/Striatal-Connectivity-based-Parcellation.
